# Whole genome doubling in adenomyosis

**DOI:** 10.1002/ctm2.1809

**Published:** 2024-08-11

**Authors:** Xiaopei Chao, Xiaojing Chen, Haiqi Su, Xiao Shang, Huanwen Wu, Yan You, Siqi Wang, Hui Li, Zhenzhen Li, Lan Zhu, Jiayan Wu, Jinghe Lang, Lei Li

**Affiliations:** ^1^ Department of Obstetrics and Gynecology Peking Union Medical College Hospital Beijing China; ^2^ National Clinical Research Center for Obstetric and Gynecologic Diseases Beijing China; ^3^ State Key Laboratory for Complex, Severe and Rare Diseases Peking Union Medical College Hospital Beijing China; ^4^ Department of Pathology Peking Union Medical College Hospital Beijing China; ^5^ Berry Oncology Corporation Beijing China; ^6^ Fujian Key Laboratory of Advanced Technology for Cancer Screening and Early Diagnosis Beijing China

Dear Editor,

Whole genome doubling (WGD) was found to be associated with an early onset of the disease and a higher response rate to endocrine therapy among adenomyosis (AM) patients. The clinical manifestation could not be explained by commonly found driver genes in AM and endometriosis (EM).

AM is one of the most common benign gynecologic diseases. The prevalence of AM has been estimated to range from 5% to 70%,^1^, ^2^, ^3^ with the highest incidence observed in women in their 40s.^4^, ^5^ Despite various conservative treatment regimens for preserving fertility in women with AM.^6^, ^7^ Currently there are no reliable strategies for predicting its occurrence and exacerbation. WGD events are defined if the ploidy of the major allele exceeds 1.5 on at least 50% of at least 11 autosomes.^8^ While WGD events have been extensively explored in tumorigenesis, metastasis, treatment, and prognosis of solid tumours, little is known about WGD in the context of AM. Thus, the aims of this study are to analyze the genomic and transcriptomic characteristics of eutopic and ectopic endometrium harvested from patients with AM and EM.

This study included a total of 79 AM patients, 22 EM patients and 20 controls (Tables S1–S3). Fresh samples of endometrium, AM lesions, and EM lesions were obtained using aseptic procedures. Paired peripheral blood samples were collected preoperatively within one day before surgery and anaesthesia. Tissue samples were sent for whole exome sequencing (WES) analysis, RNA‐sequencing, and pathological evaluation to ensure accurate histological diagnosis. The analysis of allele‐specific DNA copy number was used to determine the WGD status. B‐allele frequency values (BAF) and log *R* values were calculated based on read coverage and GC content in the same region of different specimen types. Ploidy levels were interpreted from BAF and log *R* values using the ASCAT software.^9^ WGD was detected in 33 samples from 29 patients (Figure 1A,B). The median ploidy was 3.9 (range: 3.3–4.6) in WGD samples, compared to 2.0 (range: 1.9–2.4) in non‐WGD samples (Wilcoxon rank sum test, *p *< 2.2e‐16, Figure 1C, Table S4). Ploidy levels for each autosome were determined based on the criteria of major allele ploidy being greater than 1.5 on at least 50% of at least 11 autosomes^8^ (Figure 1D). WGD samples showed a significantly higher proportion of the genome subject to loss of heterozygosity (LOH) compared to non‐WGD samples (*p *= 1.5e‐06, Figure 1E), as well as a significantly lower proportion of the genome subject to haploid LOH in WGD samples (*p *= 6.8e‐08, Figure 1E). However, within the specific histological types, the distribution of LOH varied without consistent differences (Figure S1).

**FIGURE 1:**
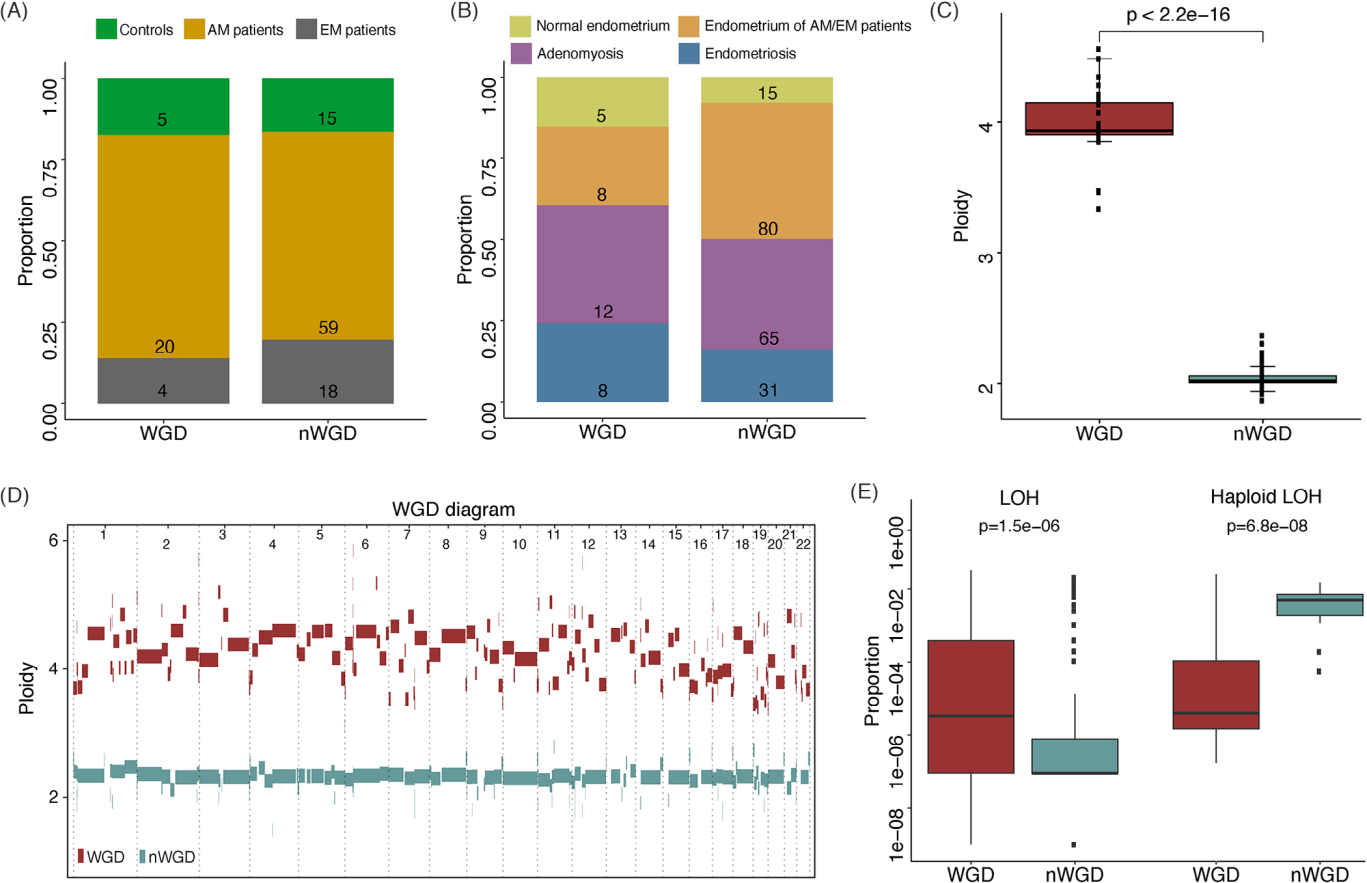
Prevalence of whole genome doubling (WGD) and non‐whole genome doubling (nWGD) genomes in adenomyosis, endometriosis and control patients. (A) Numbers and proportions of WGD and nWGD patients. (B) Numbers and proportions of WGD and nWGD samples. (C) Difference between the chromosome ploidy of WGD and nWGD samples. The coefficient of determination using the Wilcoxon rank‐sum test is indicated. (D) Overview of ploidy of WGD and nWGD samples in each autosome. (E) Proportions of the genome subject to loss of heterozygosity (LOH) and haploid LOH in WGD and nWGD samples. The coefficient of determination using the Wilcoxon rank‐sum test is indicated.

The distribution of WGD events in AM patients, EM patients, and controls was similar (*p *= .781, Figure 1A). WGD patients were significantly younger at AM onset compared to non‐WGD patients (33.5 ± 5.5 vs. 40.4 ± 6.0 years old, *p *< .001). However, WGD in EM patients and controls was not associated with disease onset age (Figure 2A). Comparison of the interval from menarche to AM onset showed that AM occurred significantly earlier in WGD patients compared to non‐WGD patients (19.5 ± 5.3 vs. 26.8 ± 6.0 years; *p *= 1.43e‐08, Figure 2B). Using an onset age of 40.5 years as the cutoff value, WGD had an area under the curve value of 0.818 (95% confidence interval [CI]: 0.724–0.913) in predicting AM early onset, with a sensitivity of 95.0% and specificity of 55.9% (Figure 2C). Univariate and multivariate analysis showed that WGD status was an independent risk factor for AM early onset (odds ratio 48.5, 95% CI: 6.454–334.811, Tables S5 and S6). In AM patients, the proportion of patients with WGD is higher in the endocrine therapy effective group (12/36 vs. 0/10, 33.3% vs. 0%, *p *= .032).

**FIGURE 2:**
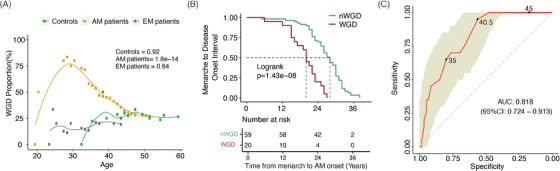
The correlation analysis between whole genome doubling (WGD) and adenomyosis onset. (A) Trend test analysis of the age at disease onset which affected WGD events. (B) The comparison of menarche to adenomyosis onset interval between WGD and non‐whole genome doubling (nWGD) groups with the Kaplan‐Meier method. (C) Receiver operating characteristic curves and associated area under the curve values in adenomyosis patients group. WGD patients (*n* =  20) were used as the positive label, and nWGD patients (*n* = 59) were used as the negative label. Three operational points (disease age thresholds) are shown that correspond with three different specificity regimes.

A total of nine somatic driver mutation genes were identified (*q *< .1) in AM patients through WES, including *KRAS*, *ARHGAP35*, *PIK3CA*, *CDC27*, *FBXW7*, *PTEN*, *PPP2R1A*, *PIK3R1* and *RRAS2* (Figure 3A). No significant differences existed in the distribution of driver genes (*p *= .07926) and single‐nucleotide variations (*p *= 1) between WGD and non‐WGD samples (Table S7). Further analysis of the co‐occurrence and mutual exclusion of driver genes showed only co‐occurrence relationships among the 9 driver genes (Figure S2A). The frequency of 33 copy number loss genes was significantly higher in WGD samples than in non‐WGD samples (*q *< .05). These differentially expressed copy number variation loss genes were involved in antigen processing and presentation, condensed chromosomes, and immune response‐regulating signalling pathways (Figure S2B). Furthermore, these copy number loss genes were extensively enriched in four WGD samples from three AM patients (38# AM, 40# EN and AM and 41# EN, Figure 3B).

**FIGURE 3:**
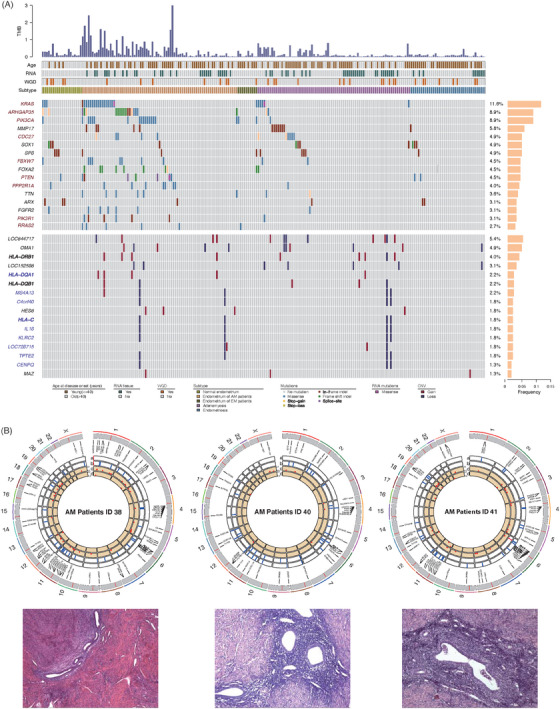
Number and allele frequency of single‐nucleotide variations (SNVs) and copy number variations (CNVs) detected in normal endometrium, endometrium of adenomyosis patients, endometrium of endometriosis patients, adenomyosis and endometriosis samples. (A) The SNVs landscape was identified by WES and RNA‐seq methods, and the copy number heterogeneity was identified by WES. SNV driver genes are in red font, and CNV differential genes between whole genome doubling (WGD) and non‐whole genome doubling (nWGD) samples are in purple font. (B) CNV amplifications (red) and deletion (blue) and the variant allele frequency (VAF) of SNVs (red dot) were exhibited from three adenomyosis patients. (a) CNV mutations detected in endometrium samples; (b) CNV mutations detected in adenomyosis samples; (c) The VAF of SNVs detected in endometrium samples. (d) The VAF of SNVs detected in adenomyosis samples.

In our study, we observed frequent *KRAS* and *PIK3CA* mutations in AM patients (*KRAS*: 19/79, 24.05%; *PIK3CA*: 15/79, 18.99%), but these mutations were less commonly found in EM patients (*KRAS*: 1/22, 4.55%; *PIK3CA*: 2/22, 9.09%) and controls (*KRAS*: 3/20, 15.00%; *PIK3CA*: 1/20, 5.00%) (Tables S8 and S9). However, no significant differences existed in the mutation frequency of *KRAS* and *PIK3CA* genes among AM patients, EM patients, and controls (*p *> .05, Tables S8 and S9). Referring to the variant allele frequency (VAF) of *KRAS* and *PIK3CA* genes, no significant differences existed among AM patients, EM patients, and controls (*p *> .05), except VAF of the *KRAS* gene between AM and EM patients (*p *= .035) (Figure S3). Moreover, *KRAS* and *PIK3CA* mutations were not associated with the age of AM onset (Figure 4A), the interval from menarche to AM onset (Figure 4B), or the response to endocrine therapy (*p *> .05, Table S10).

**FIGURE 4:**
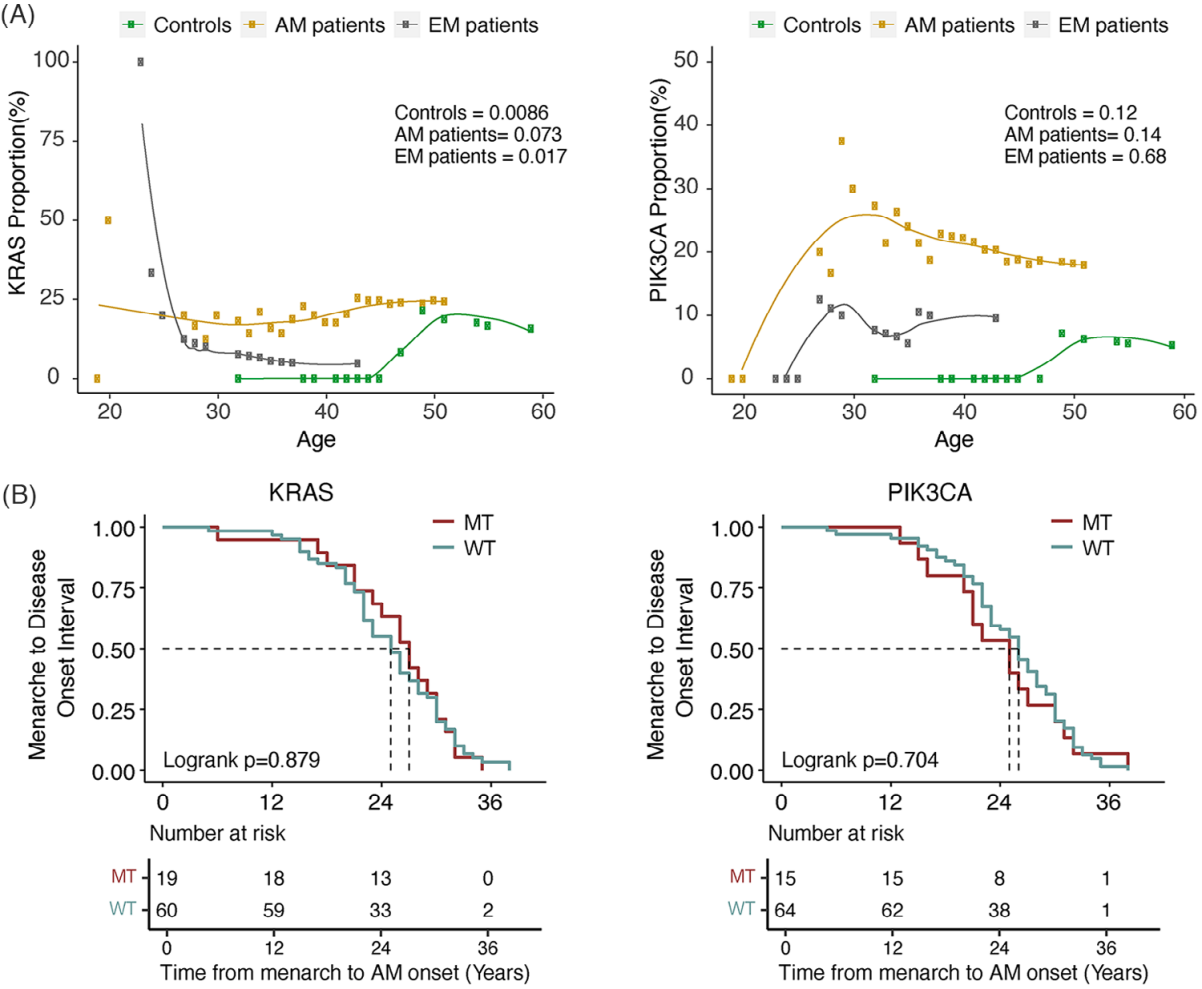
The correlation analysis of *KRAS* and *PIK3CA* mutations with age at disease onset. (A) Trend test analysis of the age at disease onset with *KRAS* (left) and *PIK3CA* (right) mutations. (B) The comparison of menarche to adenomyosis onset interval between mutation and wild type groups (*KRAS* in left and *PIK3CA* in right) with the Kaplan‐Meier method.

In summary, WGD events in AM suggest that endometrial cells may undergo endoreplication or an abortive cell cycle in response to metabolic stressors induced by estrogen. WGD events play a significant role in the early onset of AM and influence the effectiveness of endocrine therapy. There were no specific antecedent aberrant gene mutations found to be relevant to the occurrence or clinical manifestation of WGD in AM.

## AUTHOR CONTRIBUTIONS

Lei Li and Jinghe Lang conceived of the original idea for the study, interpreted the results, carried out the statistical analysis, edited the paper and were overall guarantors. Jinghe Lang and Lan Zhu obtained ethical approval, contributed to the preparation of the data set, interpreted results and contributed to drafts of the paper. Jinghe Lang, Haiqi Su, Xiao Shang, Xiaopei Chao, Xiaojing Chen, and Lan Zhu contributed to the study design, interpretation of results and commented on drafts of the paper. Siqi Wang, Hui Li, Zhenzhen Li and Jiayan Wu contributed to the interpreted data set. Huanwen Wu and Yan You conducted the pathological evaluation and reviewed the original materials. All authors have approved the final version of the manuscript.

## CONFLICT OF INTEREST STATEMENT

The authors declare no conflict of interest.

## FUNDING INFORMATION

This study is supported by the State Key Laboratory for Complex, Severe and Rare Diseases in Peking Union Medical College Hospital (No. I101301, Lei Li), by the Key Research Project of Beijing Natural Science Foundation (No. Z220013, Lei Li), by the CAMS Innovation Fund for Medical Sciences (CIFMS) (No. 2022‐I2M‐C&T‐B‐033, Lei Li), by the National High‐Level Hospital Clinical Research Funding (Nos. 2022‐PUMCH‐A‐117 [Lei Li], 2022‐PUMCH‐B‐083 [Lei Li], 2022‐PUMCH‐C‐010 [Lei Li], 2022‐PUMCH‐C‐022 [Lei Li] and 2022‐PUMCH‐D‐003 [Lan Zhu]), and by the China Postdoctoral Science Foundation (No. 2022T150066, Xiaopei Chao). The funders had no role in the study design, data collection and analysis, interpretation of data, writing of the report or decision to submit the paper for publication.

## ETHICS STATEMENT

The Institutional Review Board of Peking Union Medical College Hospital has approved this study (No. JS‐1698). The registration number is NCT03742843 (*clinicaltrials.gov*, registered on November 10, 2018). Informed consent was obtained from the subjects prior to participating in the study.

## Supporting information

Supporting Information

## Data Availability

All data from this study has been contained in the supplement file. Besides, all of the raw and processed data used in this study have been uploaded to the Genome Sequence Archive depository (https://ngdc.cncb.ac.cn/gsa) with the Accession Number (HRA003175).

## References

[1] Abbott JA . Adenomyosis and abnormal uterine bleeding (AUB‐A)‐pathogenesis, diagnosis, and management. Best Pract Res Clin Obstet Gynaecol. 2017;40:68‐81. 27810281 10.1016/j.bpobgyn.2016.09.006

[2] Guo SW . The pathogenesis of adenomyosis vis‐à‐vis endometriosis. J Clin Med. 2020;9(2):485. 32050720 10.3390/jcm9020485 PMC7073526

[3] Gordts S , Grimbizis G , Campo R . Symptoms and classification of uterine adenomyosis, including the place of hysteroscopy in diagnosis. Fertil Steril. 2018;109(3):380‐388.e381. 29566850 10.1016/j.fertnstert.2018.01.006

[4] Morassutto C , Monasta L , Ricci G , Barbone F , Ronfani L . Incidence and estimated prevalence of endometriosis and adenomyosis in Northeast Italy: a data linkage study. PLoS One. 2016;11(4):e0154227. 27101396 10.1371/journal.pone.0154227 PMC4839734

[5] Vannuccini S , Tosti C , Carmona F , et al. Pathogenesis of adenomyosis: an update on molecular mechanisms. Reprod Biomed Online. 2017;35(5):592‐601. 28693952 10.1016/j.rbmo.2017.06.016

[6] Fedele L , Bianchi S , Frontino G . Hormonal treatments for adenomyosis. Best Pract Res Clin Obstet Gynaecol. 2008;22(2):333‐339. 17765017 10.1016/j.bpobgyn.2007.07.006

[7] Chao X , Song X , Wu H , You Y , Li L , Lang J . Adjuvant therapy in conservative surgery for adenomyosis. Int J Gynaecol Obstet. 2021;154(1):119‐126. 33368241 10.1002/ijgo.13573

[8] Priestley P , Baber J , Lolkema MP , et al. Pan‐cancer whole‐genome analyses of metastatic solid tumours. Nature. 2019;575(7781):210‐216. 31645765 10.1038/s41586-019-1689-y PMC6872491

[9] Ross EM , Haase K , Van Loo P , Markowetz F . Allele‐specific multi‐sample copy number segmentation in ASCAT. Bioinformatics. 2021;37(13):1909‐1911. 32449758 10.1093/bioinformatics/btaa538 PMC8317109

