# Supplementary material for: Whole genome doubling in adenomyosis
Source: Clin Transl Med. 2024 Aug 11;14(8):e1809. doi: 10.1002/ctm2.1809 (PMC11317496; doi:10.1002/ctm2.1809)
Supplement: Supplementary file 1 — Supporting Information [file CTM2-14-e1809-s001.docx]

**Supplementary Files**

**Index**

[Supplementary Table 1. The epidemiology and clinicopathological data of the 79 patients with adenomyosis. 2](#_Toc172791350)

[Supplementary Table 2. The epidemiology and clinicopathological data of the 22 patients with endometriosis. 4](#_Toc172791351)

[Supplementary Table 3. The epidemiology and clinicopathological data of the 20 controls. 6](#_Toc172791352)

[Supplementary Table 4. The prevalence of WGD events 7](#_Toc172791353)

[Supplementary Table 5. Univariate analysis of the early onset of adenomyosis. 12](#_Toc172791354)

[Supplementary Table 6. Multivariate analysis of the early onset of adenomyosis. 14](#_Toc172791355)

[Supplementary Table 7. The sample distribution of SNV and driver genes. 15](#_Toc172791356)

[Supplementary Table 8. The distribution of patients with *KRAS* mutation. 16](#_Toc172791357)

[Supplementary Table 9. The distribution of patients with *PIK3CA* mutation. 17](#_Toc172791358)

[Supplementary Table 10. The relationship between *KRAS* and *PIK3CA* mutations and effectiveness of endocrine therapy. 18](#_Toc172791359)

[Supplementary Figure 1. Proportions of the genome subject to loss of heterozygosity (LOH) and haploid LOH in WGD and nWGD samples. 19](#_Toc172791360)

[Supplementary Figure 2. The mutual exclusion and co-occurrence relationship between driver genes (A), and GO and KEGG pathway enrichment analysis of 33 differential CNV loss genes performed by Metascape (B). 20](#_Toc172791361)

[Supplementary Figure 3. Comparison of variant allele frequency (VAF) of *KRAS* and *PIK3CA* genes among AM patients, EM patients and controls groups. For multiple-lesion patients, variants detected in more than one sample, VAF take the maximum as the measured value based on WES. 21](#_Toc172791362)

# Supplementary Table 1. The epidemiology and clinicopathological data of the 79 patients with adenomyosis.

|  | Total (n=79) | WGD group (n=20) | non-WGD group (n=59) | *P* value |
| --- | --- | --- | --- | --- |
| Age at menarche (years), mean±SD | 13.7 ± 1.3 | 14.0 ± 1.6 | 13.6 ± 1.3 | 0.355 |
| Age at surgery (years), mean±SD | 45.4 ± 4.6 | 45.1 ± 5.4 | 45.5 ± 4.3 | 0.711 |
| Age at disease onset (years), mean±SD | 38.7 ± 6.6 | 33.5 ± 5.5 | 40.4 ± 6.0 | **<0.001** |
| Age at disease onset before 30, n (%) | 8 (10.1) | 5 (25.0) | 3 (5.1) | **0.022** |
| Age at disease onset before 40, n (%) | 34 (43.0) | 16 (8.0) | 18 (30.5) | **<0.001** |
| Age at disease onset before 50, n (%) | 61 (77.2) | 16 (8.0) | 45 (76.3) | 0.497 |
| Menarche to disease onset interval (month), mean±SD | 299.5 ± 79.7 | 234.0 ± 63.3 | 321.8 ± 72.4 | **<0.001** |
| Height (cm), median (range) | 160 (150-170) | 160 (153-168) | 160 (150-170) | 0.812 |
| Body weight (kg), median (range) | 63.5 (45-99) | 63.0 (45-87) | 63.5 (45-99) | 0.882 |
| BMI (kg/m^2^), median (range) | 24.2 (18-34) | 24.9 (18-32) | 24.2 (18-34) | 0.913 |
| Gravidity (times),median (range) | 2 (0-7) | 2 (0-6) | 3 (0-7) | 0.545 |
| Parity (times),median (range) | 1 (0-3) | 1 (0-1) | 1 (0-3) | 0.698 |
| Menstrual cycle, n (%) |  |  |  | 0.399 |
| Follicular phase | 19 (24.1) | 4 (20.0) | 15 (25.4) |  |
| Luteal phase | 21 (26.6) | 3 (15.0) | 18 (30.5) |  |
| Under treatment | 31 (39.2) | 10 (50.0) | 21 (35.6) |  |
| Not available | 8 (10.1) | 3 (15.0) | 5 (8.5) |  |
| Dysmenorrhea, n (%) | 65 (82.3) | 16 (80.0) | 49 (83.1) | 0.744 |
| Vaginal bleeding, n (%) | 55 (69.6) | 13 (65.0) | 42 (71.2) | 0.603 |
| Hormone therapy before surgery, n (%) | 47 (59.5) | 13 (65.0) | 34 (57.6) | 0.562 |
| Hormone therapy regimens, n (%) |  |  |  | 0.308 |
| GnRHa | 30 (38.0) | 8 (40.0) | 22 (37.3) | 0.929 |
| Mirena | 10 (12.7) | 1 (5.0) | 9 (15.3) | 0.438 |
| GnRHa plus Mirena | 10 (12.7) | 4 (20.0) | 6 (10.2) | 0.263 |
| Effectiveness of hormone therapy, n (%) |  |  |  |  |
| Improvement of painess | 16/27 (59.3) | 7/8 (87.5) | 9/19 (47.4) | 0.062 |
| Reduction of uterine volume | 25/28 (89.3) | 7/7 (100.0) | 18/21 (85.7) | 0.551 |
| Improvement of painess or bleeding | 18/28 (64.3) | 7/8 (87.5) | 11/20 (55.0) | 0.194 |
| Effectiveness of treatment | 36/46 (78.3) | 12/12 (100.0) | 24/34 (70.6) | **0.044** |
| History of cesarean section, n (%) | 31 (39.2) | 6 (30.0) | 25 (42.4) | 0.327 |
| History of diagnostic curettage, n (%) | 28 (35.4) | 4 (20.0) | 24 (40.7) | 0.112 |
| Concurrent leiomyoma (Pathology), n (%) | 46 (58.2) | 11 (55.0) | 35 (59.3) | 0.735 |
| Endometrial poylp (Pathology), n (%) | 9 (11.4) | 3 (15.0) | 6 (10.2) | 0.685 |
| Endometriosis (Pathology), n (%) | 34 (43.0) | 12 (60.0) | 22 (37.3) | 0.076 |
| Ovarian type | 18 (22.8) | 7 (35.0) | 11 (18.6) | 0.132 |
| DIE | 16 (20.3) | 6 (30.0) | 10 (16.9) | 0.209 |
| Peritoneal type | 19 (24.1) | 5 (25.0) | 14 (23.7) | 0.908 |

Abbreviations: BMI, Body mass index; DIE, deep invasive endometriosis; GnRHa, gonadotrophin releasing hormone agonist; SD, standard deviation.

# Supplementary Table 2. The epidemiology and clinicopathological data of the 22 patients with endometriosis.

|  | Total (n=22) | WGD group (n=4) | non-WGD group (n=18) | *P* value |
| --- | --- | --- | --- | --- |
| Age at menarche (years), mean±SD | 12.7 ± 0.8 | 13.3 ± 0.5 | 12.6 ± 0.8 | 0.135 |
| Age at surgery (years), mean±SD | 32.8 ± 6.2 | 33.0 ± 6.2 | 32.7 ± 6.4 | 0.939 |
| Age at disease onset (years), mean±SD | 31.4 ± 6.4 | 30.1 ± 5.3 | 31.6 ± 6.7 | 0.825 |
| Age at disease onset before 30, n (%) | 10 (45.5) | 2 (50.0) | 8 (44.4) | 0.999 |
| Age at disease onset before 40, n (%) | 20 (90.9) | 4 (100.0) | 16 (88.9) | 0.999 |
| Age at disease onset before 50, n (%) | 22 (100.0) | 4 (100.0) | 18 (100.0) | - |
| Menarche to disease onset interval (month), mean±SD | 224.2 ± 75.8 | 210.0 ± 60.8 | 227.3 ± 80.0 | 0.690 |
| Height (cm), median (range) | 163 (155-173) | 165 (155-169) | 163 (158-173) | 0.967 |
| Body weight (kg), median (range) | 58 (45-80) | 54 (52-65) | 60 (45-80) | 0.485 |
| BMI (kg/m^2^), median (range) | 21.7 (17-32) | 20.7 (20-23) | 21.9 (17-32) | 0.483 |
| Gravidity (times),median (range) | 1 (0-3) | 2 (0-2) | 1 (0-3) | 0.212 |
| Parity (times),median (range) | 0.5 (0-2) | 1 (0-1) | 0 (0-2) | 0.610 |
| Menstrual cycle, n (%) |  |  |  | 0.090 |
| Follicular phase | 11 (50.0) | 0 (0) | 11 (61.1) |  |
| Luteal phase | 11 (50.0) | 4 (100.0) | 7 (38.9) |  |
| Under treatment | 0 (0) | 0 (0) | 0 (0) |  |
| Not available | 0 (0) | 0 (0) | 0 (0) |  |
| Dysmenorrhea, n (%) | 8 (36.4) | 0 (0) | 8 (44.4) | 0.254 |
| Vaginal bleeding, n (%) | 2 (0.1) | 0 (0) | 2 (11.1) | 0.999 |
| Hormone therapy before surgery, n (%) | 1 (4.5) | 1 (25.0) | 0 (0) | 0.182 |
| History of cesarean section, n (%) | 7 (31.8) | 2 (50.0) | 5 (27.8) | 0.565 |
| History of diagnostic curettage, n (%) | 7 (31.8) | 2 (50.0) | 5 (27.8) | 0.565 |
| Concurrent leiomyoma (Pathology), n (%) | 0 (0) | 0 (0) | 0 (0) | - |
| Endometrial poylp (Pathology), n (%) | 2 (0.1) | 0 (0) | 2 (11.1) | 0.999 |

Abbreviations: BMI, Body mass index; NA, not available; SD, standard deviation.

# Supplementary Table 3. The epidemiology and clinicopathological data of the 20 controls.

|  | Total (n=20) | WGD group (n=5) | non-WGD group (n=15) | *P* value |
| --- | --- | --- | --- | --- |
| Age at menarche (years), mean±SD | 13.8 ± 1.2 | 13.6 ± 0.5 | 13.9 ± 1.4 | 0.678 |
| Age at surgery (years), mean±SD | 47.2 ± 8.0 | 45.2 ± 6.4 | 47.8 ± 8.6 | 0.545 |
| Age at disease onset (years), mean±SD | 47.0 ± 8.1 | 45.5 ± 7.3 | 47.4 ± 8.5 | 0.686 |
| Age at disease onset before 30, n (%) | 0 (0) | 0 (0) | 0 (0) | - |
| Age at disease onset before 40, n (%) | 4 (20.0) | 1 (20.0) | 3 (20.0) | 0.999 |
| Age at disease onset before 50, n (%) | 14 (70.0) | 4 (80.0) | 10 (66.7) | 0.999 |
| Menarche to disease onset interval (month), mean±SD | 388.5 ± 91.6 | 344.0 ± 48.5 | 398.8 ± 97.4 | 0.368 |
| Height (cm), median (range) | 161 (155-168) | 164 (156-168) | 160 (155-168) | 0.145 |
| Body weight (kg), median (range) | 57.5 (44-77) | 55.8 (53-77) | 58.0 (44-66) | 0.628 |
| BMI (kg/m^2^), median (range) | 22.6 (17-29) | 21.3 (20-29) | 22.9 (17-27) | 0.886 |
| Gravidity (times),median (range) | 2.5 (1-6) | 4 (1-6) | 2 (1-6) | 0.425 |
| Parity (times),median (range) | 1.5 (1-4) | 2 (1-2) | 1 (1-4) | 0.774 |
| Menstrual cycle, n (%) |  |  |  | 0.773 |
| Follicular phase | 9 (45.0) | 2 (40.0) | 7 (46.7) |  |
| Luteal phase | 4 (20.0) | 2 (40.0) | 2 (13.3) |  |
| Not available | 7 (35.0) | 1 (20.0) | 6 (40.0) |  |
| History of cesarean section, n (%) | 6 (30.0) | 4 (80.0) | 2 (13.3) | **0.014** |
| History of diagnostic curettage, n (%) | 11 (55.0) | 4 (80.0) | 7 (46.7) | 0.319 |
| Concurrent leiomyoma (Pathology), n (%) | 8 (40.0) | 2 (40.0) | 6 (40.0) | 0.999 |
| Endometrial poylp (Pathology), n (%) | 1 (5.0) | 1 (20.0) | 0 (0) | 0.250 |

Abbreviations: BMI, Body mass index; SD, standard deviation.

# Supplementary Table 4. The prevalence of WGD events

| **Sample** | **Type** | **WGD** | **TMB** | **Age** | **Ploidy** |
| --- | --- | --- | --- | --- | --- |
| C2200663 | EN_AM | WGD | 0.88 | 39 | 3.902583971 |
| C2200677 | AM | WGD | 0.32 | 40 | 3.917235351 |
| C2200664 | AM | WGD | 0.34 | 39 | 3.921495879 |
| C2106115 | AM | WGD | 0.05 | 33 | 3.90391615 |
| C2200607 | EM | WGD | 0 | 39 | 3.933857799 |
| C2106159 | EM | WGD | 0.07 | 27 | 3.891656859 |
| C2106162 | EM | WGD | 0.1 | 24 | 3.911437047 |
| C2106189 | EM | WGD | 0.05 | 35 | 3.903257716 |
| C2200638 | EM | WGD | 0.1 | 29 | 3.933424094 |
| C2106167 | EN_EM | WGD | 0 | 35 | 3.89179438 |
| C2200625 | EN | WGD | 0.07 | 38 | 3.923915171 |
| C2200658 | EN | WGD | 0.15 | 44 | 3.848625084 |
| C2200678 | EN | WGD | 0.12 | 42 | 3.903957464 |
| C2200681 | EN | WGD | 0.75 | 55 | 3.911033697 |
| C2106124 | EN | WGD | 0.27 | 47 | 3.945457565 |
| C1803376 | EN_AM | WGD | 0.46 | 30 | 4.557691652 |
| C1803291 | EN_AM | WGD | 0.15 | 36 | 3.98541932 |
| C1803308 | EN_AM | WGD | 0.05 | 33 | 4.279516261 |
| C1803274 | EN_AM | WGD | 0.19 | 41 | 3.472107863 |
| C1803268 | EN_AM | WGD | 0.58 | 29 | 4.161468587 |
| C1803264 | EN_AM | WGD | 0.02 | 35 | 4.209352253 |
| C1902964 | EN_AM | nWGD | 0.51 | 27 | 2.014552239 |
| C1803357 | AM | WGD | 0.05 | 28 | 4.174554883 |
| C1803316 | AM | WGD | 0.1 | 34 | 4.146441794 |
| C1803385 | AM | WGD | 0 | 30 | 3.954324587 |
| C1803321 | AM | WGD | 0.56 | 40 | 3.943738657 |
| C1803301 | AM | WGD | 0.02 | 35 | 4.13267738 |
| C1803294 | AM | WGD | 0.05 | 35 | 4.17501202 |
| C1803280 | AM | WGD | 0.05 | 20 | 3.334012082 |
| C1803273 | AM | WGD | 0 | 41 | 3.465105901 |
| C1803263 | AM | WGD | 0 | 35 | 4.07155077 |
| C1803374 | EM | WGD | 0.07 | 30 | 4.482701026 |
| C1803341 | EM | WGD | 0.24 | 38 | 4.34334822 |
| C1803324 | EM | WGD | 0.02 | 27 | 3.948876291 |
| C2106147 | EN_AM | nWGD | 0.12 | 33 | 2.03489034 |
| C2106164 | EN_AM | nWGD | 0.53 | 43 | 2.015326683 |
| C2106168 | EN_AM | nWGD | 0.36 | 29 | 2.030835413 |
| C2200645 | EN_AM | nWGD | 1.6 | 37 | 2.007351221 |
| C2200666 | EN_AM | nWGD | 0.75 | 38 | 2.000437386 |
| C2200676 | EN_AM | nWGD | 0.17 | 40 | 2.00605103 |
| C2106114 | EN_AM | nWGD | 0.07 | 35 | 2.003592262 |
| C2106180 | EN_AM | nWGD | 0.19 | 46 | 2.004373849 |
| C2200598 | EN_AM | nWGD | 0.07 | 44 | 2.044448127 |
| C2200619 | EN_AM | nWGD | 0.36 | 34 | 2.072377989 |
| C2200679 | EN_AM | nWGD | 1.24 | 34 | 2.055067409 |
| C2200602 | EN_AM | nWGD | 0.24 | 32 | 2.008218903 |
| C2106132 | EN_AM | nWGD | 0 | 41 | 2.049184126 |
| C2106158 | EN_AM | nWGD | 0.34 | 19 | 2.131676362 |
| C2106181 | EN_AM | nWGD | 2.99 | 40 | 2.084790849 |
| C2106096 | EN_AM | nWGD | 0.05 | 49 | 2.048028817 |
| C2106099 | EN_AM | nWGD | 0.02 | 34 | 1.86750312 |
| C2106116 | EN_AM | nWGD | 0 | 33 | 2.057462737 |
| C2200615 | EN_AM | nWGD | 0.07 | 39 | 2.007106733 |
| C2200642 | EN_AM | nWGD | 0.36 | 39 | 2.005928258 |
| C2106148 | AM | nWGD | 0.15 | 33 | 2.139695295 |
| C2106156 | AM | nWGD | 0.02 | 43 | 2.040992271 |
| C2106169 | AM | nWGD | 0.15 | 29 | 2.012938395 |
| C2200644 | AM | nWGD | 0.05 | 37 | 2.041901961 |
| C2200667 | AM | nWGD | 0.46 | 38 | 2.079738914 |
| C2106113 | AM | nWGD | 0.05 | 35 | 2.032689646 |
| C2106186 | AM | nWGD | 0.1 | 46 | 2.002532228 |
| C2200597 | AM | nWGD | 0.36 | 44 | 2.062504143 |
| C2200620 | AM | nWGD | 0.17 | 34 | 2.008856438 |
| C2200680 | AM | nWGD | 0.8 | 34 | 2.002045744 |
| C2200604 | AM | nWGD | 0.02 | 32 | 2.029233905 |
| C2106134 | AM | nWGD | 0.05 | 41 | 2.036672955 |
| C2106153 | AM | nWGD | 0.07 | 19 | 2.062460755 |
| C2106185 | AM | nWGD | 0.12 | 40 | 2.179956108 |
| C2106097 | AM | nWGD | 0.05 | 49 | 2.002234674 |
| C2106100 | AM | nWGD | 0.24 | 34 | 2.11078631 |
| C2200616 | AM | nWGD | 0.05 | 39 | 2.100429894 |
| C2106131 | EM | nWGD | 0 | 41 | 2.020785754 |
| C2106157 | EM | nWGD | 0 | 19 | 2.222930989 |
| C2106182 | EM | nWGD | 0.1 | 40 | 2.080966948 |
| C2106098 | EM | nWGD | 0.19 | 49 | 2.008938697 |
| C2106112 | EM | nWGD | 0.02 | 33 | 2.001381774 |
| C2106092 | EM | nWGD | 0 | 33 | 2.003537541 |
| C2106095 | EM | nWGD | 0.1 | 37 | 2.106592097 |
| C2106102 | EM | nWGD | 0 | 32 | 2.004820868 |
| C2106125 | EM | nWGD | 0.05 | 25 | 2.026278837 |
| C2106129 | EM | nWGD | 0.07 | 28 | 2.008014248 |
| C2106126 | EM | nWGD | 0.05 | 27 | 2.045607939 |
| C2106146 | EM | nWGD | 0.05 | 27 | 2.005173786 |
| C2106154 | EM | nWGD | 0.1 | 25 | 2.00496952 |
| C2106187 | EM | nWGD | 0.05 | 34 | 2.002045935 |
| C2106155 | EM | nWGD | 0.12 | 23 | 2.008381003 |
| C2106142 | EM | nWGD | 0.07 | 35 | 2.103694167 |
| C2106151 | EM | nWGD | 0.12 | 32 | 2.000740491 |
| C2200603 | EM | nWGD | 0.44 | 27 | 2.055420402 |
| C2200617 | EM | nWGD | 0.07 | 36 | 2.0025476 |
| C2200614 | EM | nWGD | 0.12 | 48 | 2.014612264 |
| C2200606 | EM | nWGD | 0.05 | 32 | 2.114127369 |
| C2106172 | EM | nWGD | 0 | 43 | 2.153915242 |
| C2106133 | EM | nWGD | 0 | 24 | 2.037414966 |
| C2106141 | EN_EM | nWGD | 0 | 35 | 2.226833053 |
| C2106145 | EN_EM | nWGD | 0 | 32 | 2.004611242 |
| C2200609 | EN_EM | nWGD | 0.17 | 27 | 2.004365648 |
| C2200618 | EN_EM | nWGD | 0.34 | 36 | 2.043562829 |
| C2200613 | EN_EM | nWGD | 0.05 | 48 | 2.007751427 |
| C2200608 | EN_EM | nWGD | 0.34 | 32 | 2.214283344 |
| C2106173 | EN_EM | nWGD | 0.07 | 43 | 2.03496108 |
| C2106137 | EN_EM | nWGD | 0.19 | 24 | 2.016606643 |
| C2200637 | EN_EM | nWGD | 0 | 29 | 1.918581485 |
| C2106179 | EN | nWGD | 0.29 | 49 | 2.082577848 |
| C2200624 | EN | nWGD | 0.34 | 39 | 2.002438019 |
| C2200641 | EN | nWGD | 0.1 | 44 | 2.033232124 |
| C2200670 | EN | nWGD | 0.44 | 51 | 2.02666001 |
| C2106108 | EN | nWGD | 0.27 | 43 | 2.004068402 |
| C2106140 | EN | nWGD | 0.05 | 42 | 2.024774775 |
| C2200627 | EN | nWGD | 0.12 | 41 | 2.024359359 |
| C2106188 | EN | nWGD | 0.02 | 32 | 1.918901976 |
| C2106130 | EN | nWGD | 0.29 | 49 | 2.008868593 |
| C2106121 | EN | nWGD | 0.07 | 54 | 2.010527358 |
| C2106106 | EN | nWGD | 0.05 | 45 | 2.022851859 |
| C2200665 | EN | nWGD | 0.22 | 59 | 2.01749632 |
| C2200661 | EN | nWGD | 0.07 | 65 | 2.046503892 |
| C2200626 | EN | nWGD | 0.07 | 39 | 2.072722394 |
| C2200684 | EN | nWGD | 0.66 | 51 | 2.022563356 |
| C1803358 | EN_AM | nWGD | 0 | 28 | 2.00525497 |
| C1803340 | EN_AM | nWGD | 0.46 | 43 | 2.044092747 |
| C1803343 | EN_AM | nWGD | 0.19 | 38 | 2.107389363 |
| C1803326 | EN_AM | nWGD | 0.12 | 27 | 2.000911116 |
| C1803317 | EN_AM | nWGD | 0 | 34 | 2.002708076 |
| C1803272 | EN_AM | nWGD | 0.05 | 43 | 2.009534473 |
| C1803251 | EN_AM | nWGD | 0 | 51 | 2.004544833 |
| C1803248 | EN_AM | nWGD | 0.1 | 40 | 2.056539101 |
| C1902981 | EN_AM | nWGD | 0.29 | 36 | 2.210776177 |
| C1803400 | EN_AM | nWGD | 0 | 44 | 2.028991822 |
| C1803397 | EN_AM | nWGD | 0.05 | 41 | 2.00003354 |
| C1803395 | EN_AM | nWGD | 0.39 | 41 | 2.006029949 |
| C1803388 | EN_AM | nWGD | 1.51 | 50 | 2.035627135 |
| C1803384 | EN_AM | nWGD | 0.05 | 30 | 2.00462451 |
| C1803381 | EN_AM | nWGD | 0.36 | 42 | 2.001554895 |
| C1803378 | EN_AM | nWGD | 0.05 | 35 | 2.004354186 |
| C1803372 | EN_AM | nWGD | 0.1 | 39 | 2.001812506 |
| C1803369 | EN_AM | nWGD | 0.17 | 42 | 1.982090754 |
| C1803356 | EN_AM | nWGD | 0.05 | 37 | 2.00384216 |
| C1803352 | EN_AM | nWGD | 0.12 | 47 | 2.013471395 |
| C1803346 | EN_AM | nWGD | 0.88 | 47 | 2.037719776 |
| C1803348 | EN_AM | nWGD | 0.17 | 42 | 2.015732751 |
| C1803337 | EN_AM | nWGD | 1.8 | 44 | 2.009752641 |
| C1803332 | EN_AM | nWGD | 0.53 | 35 | 2.00274056 |
| C1803329 | EN_AM | nWGD | 2.41 | 41 | 2.038619252 |
| C1803322 | EN_AM | nWGD | 0.97 | 40 | 2.002755932 |
| C1803314 | EN_AM | nWGD | 0 | 44 | 2.019117101 |
| C1803306 | EN_AM | nWGD | 0.24 | 46 | 1.992936359 |
| C1803302 | EN_AM | nWGD | 0.05 | 35 | 2.004698352 |
| C1803300 | EN_AM | nWGD | 0.02 | 34 | 2.009267492 |
| C1803295 | EN_AM | nWGD | 1.56 | 35 | 2.086132289 |
| C1803293 | EN_AM | nWGD | 0.73 | 46 | 2.012031591 |
| C1803290 | EN_AM | nWGD | 0.61 | 45 | 2.008602223 |
| C1803283 | EN_AM | nWGD | 0.51 | 44 | 2.002909166 |
| C1803281 | EN_AM | nWGD | 0.85 | 20 | 2.08523628 |
| C1803278 | EN_AM | nWGD | 0.05 | 45 | 1.94575681 |
| C1803261 | EN_AM | nWGD | 0.39 | 43 | 2.017263058 |
| C1803258 | EN_AM | nWGD | 1.19 | 41 | 2.154193702 |
| C1803255 | EN_AM | nWGD | 0.24 | 37 | 2.303417513 |
| C1803244 | EN_AM | nWGD | 0 | 43 | 2.023827303 |
| C1902740 | EN_AM | nWGD | 0.46 | 38 | 2.11684554 |
| C1902950 | EN_AM | nWGD | 0.02 | 36 | 2.130390699 |
| C1902954 | EN_AM | nWGD | 0.88 | 53 | 2.067531426 |
| C1902956 | EN_AM | nWGD | 0 | 44 | 2.074910502 |
| C1902968 | EN_AM | nWGD | 1.17 | 47 | 2.001827121 |
| C1902960 | EN_AM | nWGD | 0.29 | 45 | 2.001537176 |
| C1902972 | EN_AM | nWGD | 0.58 | 39 | 2.030657428 |
| C1902976 | EN_AM | nWGD | 0 | 45 | 2.053932584 |
| C1902978 | EN_AM | nWGD | 0.29 | 40 | 2.019128763 |
| C1902984 | EN_AM | nWGD | 0.19 | 37 | 2.015663422 |
| C1803375 | AM | nWGD | 0.39 | 30 | 2.044448989 |
| C1803339 | AM | nWGD | 0.53 | 43 | 2.056981928 |
| C1803342 | AM | nWGD | 0 | 38 | 2.141933956 |
| C1803325 | AM | nWGD | 0.02 | 27 | 2.002159681 |
| C1803287 | AM | nWGD | 0.02 | 36 | 2.056847892 |
| C1803271 | AM | nWGD | 0.05 | 43 | 2.009432682 |
| C1803250 | AM | nWGD | 0.02 | 51 | 2.152644888 |
| C1803247 | AM | nWGD | 0 | 40 | 2.233178037 |
| C1902982 | AM | nWGD | 0.22 | 36 | 2.21369763 |
| C1803399 | AM | nWGD | 0 | 44 | 2.037538544 |
| C1803396 | AM | nWGD | 0.07 | 41 | 2.000268321 |
| C1803394 | AM | nWGD | 0.07 | 41 | 2.001607986 |
| C1803387 | AM | nWGD | 0 | 50 | 2.044028446 |
| C1803382 | AM | nWGD | 0.02 | 42 | 2.005374527 |
| C1803379 | AM | nWGD | 0.05 | 35 | 2.003877947 |
| C1803371 | AM | nWGD | 0 | 39 | 2.005739603 |
| C1803368 | AM | nWGD | 0.53 | 42 | 2.007378341 |
| C1803355 | AM | nWGD | 0.02 | 37 | 2.058878505 |
| C1803351 | AM | nWGD | 0.07 | 47 | 2.032449093 |
| C1803345 | AM | nWGD | 0.05 | 47 | 2.021125801 |
| C1803347 | AM | nWGD | 0 | 42 | 2.017222858 |
| C1803336 | AM | nWGD | 0.56 | 44 | 2.008504033 |
| C1803331 | AM | nWGD | 0.12 | 35 | 1.95743673 |
| C1803328 | AM | nWGD | 0 | 41 | 2.002722506 |
| C1803313 | AM | nWGD | 0.02 | 44 | 2.008781707 |
| C1803307 | AM | nWGD | 0.02 | 33 | 2.006934955 |
| C1803305 | AM | nWGD | 0.02 | 46 | 2.017607917 |
| C1803299 | AM | nWGD | 0 | 34 | 2.07735449 |
| C1803292 | AM | nWGD | 0.12 | 46 | 2.005478071 |
| C1803289 | AM | nWGD | 0.36 | 45 | 2.054932548 |
| C1803282 | AM | nWGD | 0.07 | 44 | 2.012081594 |
| C1803277 | AM | nWGD | 0.07 | 45 | 2.026831433 |
| C1803267 | AM | nWGD | 0.1 | 29 | 2.014339259 |
| C1803260 | AM | nWGD | 0.15 | 43 | 2.066673463 |
| C1803257 | AM | nWGD | 0.05 | 41 | 2.127254815 |
| C1803254 | AM | nWGD | 0 | 37 | 2.365865111 |
| C1803243 | AM | nWGD | 0.46 | 43 | 2.047518838 |
| C1902741 | AM | nWGD | 0.66 | 38 | 2.142145813 |
| C1902951 | AM | nWGD | 0.7 | 36 | 2.077396242 |
| C1902955 | AM | nWGD | 0.05 | 53 | 2.009360792 |
| C1902957 | AM | nWGD | 0.02 | 44 | 2.044832547 |
| C1902965 | AM | nWGD | 0 | 27 | 2.017299864 |
| C1902969 | AM | nWGD | 0.78 | 47 | 1.941366022 |
| C1902961 | AM | nWGD | 0 | 45 | 2.01015873 |
| C1902973 | AM | nWGD | 0.05 | 39 | 2.050599966 |
| C1902977 | AM | nWGD | 0.02 | 45 | 2.066523463 |
| C1902979 | AM | nWGD | 0.75 | 40 | 2.059441719 |
| C1902985 | AM | nWGD | 0.12 | 37 | 2.015326574 |
| C1803354 | EM | nWGD | 0.15 | 28 | 2.007917027 |
| C1803335 | EM | nWGD | 0.05 | 43 | 2.047548291 |
| C1803315 | EM | nWGD | 0 | 34 | 2.000548471 |
| C1803286 | EM | nWGD | 0.05 | 36 | 2.022524636 |
| C1803270 | EM | nWGD | 0 | 43 | 2.008211183 |
| C1803253 | EM | nWGD | 0.02 | 51 | 2.12072854 |
| C1803246 | EM | nWGD | 0.02 | 40 | 2.088552413 |
| C1902986 | EM | nWGD | 0 | 36 | 2.090432242 |

# Supplementary Table 5. Univariate analysis of the early onset of adenomyosis.

|  | Total (n=79) | Early onset (n=34) | Late onset(n=45) | *P* value | OR (95% CI) |
| --- | --- | --- | --- | --- | --- |
| WGD existing, n(%) | 20 (25.3) | 16 (47.1) | 4 (8.9) | **<0.001** | 9.111 (2.669-31.104) |
| Age at menarche (years), mean±SD | 13.7 ± 1.3 | 13.7 ± 1.3 | 13.7 ± 1.4 | 0.986 |  |
| Age at surgery (years), mean±SD | 45.4 ± 4.6 | 44.0 ± 4.8 | 46.5± 4.1 | **0.017** |  |
| Age at disease onset (years), mean±SD | 38.7 ± 6.6 | 34.2 ± 4.3 | 42.1 ± 6.0 | **<0.001** |  |
| Menarche to disease onset interval (month), mean±SD | 299.5 ± 79.7 | 245.3 ± 54.6 | 340.5 ± 70.8 | **<0.001** |  |
| Height (cm), median (range) | 160.6 ± 4.6 | 161.3 ± 4.8 | 160.1 ± 4.4 | 0.244 | 1.062 (0.960-1.174) |
| Body weight (kg), median (range) | 63.2± 10.9 | 64.8 ± 10.8 | 62.1 ± 11.0 | 0.281 | 1.023 (0.981-1.067) |
| BMI (kg/m^2^), median (range) | 24.4 ± 3.6 | 24.8 ± 3.7 | 24.2 ± 3.5 | 0.401 | 1.056 (0.931-1.197) |
| Gravidity (times), median (range) | 2 (0-7) | 2 (0-6) | 3 (0-7) | 0.073 | 0.771 (0.579-1.028) |
| Parity (times), median (range) | 1(0-3) | 1 (0-3) | 1 (0-2) | 0.795 | 0.884 (0.355-2.205) |
| Menstrual cycle, n (%) |  |  |  |  |  |
| Follicular phase | 19 (24.1) | 7 (20.6) | 12 (26.7) | 0.916 |  |
| Luteal phase | 21 (26.6) | 9 (26.5) | 12 (26.7) | 0.698 | 1.286 (0.361-4.584) |
| Under treatment | 31 (39.2) | 14 (41.2) | 17 (37.8) | 0.564 | 1.412 (0.438-4.549) |
| Not available | 8 (10.1) | 4 (11.8) | 4 (8.9) | 0.527 | 1.714 (0.323-9.109) |
| Dysmenorrhea, n (%) | 65 (82.3) | 29 (85.3) | 36 (80.0) | 0.542 | 0.690 (0.208-2.284) |
| Vaginal bleeding, n (%) | 55 (69.6) | 25 (3.5) | 30 (66.7) | 0.511 | 0.720 (0.270-1.923) |
| Hormone therapy before surgery, n (%) | 47 (59.5) | 21(61.8) | 26 (57.8) | 0.721 | 0.847 (0.341-2.105) |
| Progesterone therapy regimens, n (%) |  |  |  |  |  |
| GnRHa | 30 (38.0) | 13 (38.2) | 17 (37.8) | 0.967 | 0.981 (0.392-2.455) |
| Mirena | 10 (12.7) | 4 (11.8) | 6 (13.3) | 0.558 | 1.154 (0.299-4.459) |
| GnRHa plus Mirena | 10 (12.7) | 6 (17.6) | 4 (8.9) | 0.206 | 0.455 (0.118-1.762) |
| Improvement of painess, n (%) | 16/27 (59.3) | 10/15 (66.7) | 6/12 (50.0) | 0.381 |  |
| Improvement of bleeding, n (%) | 7/12 (58.3) | 5/8 (62.5) | 2/4 (50.0) | 0.576 |  |
| Reduction of uterine volume, n (%) | 25/28 (89.3) | 11/12 (91.7) | 14/16 (87.5) | 0.611 |  |
| Improvement of painess or bleeding, n (%) | 18/28 (64.3) | 11/15 (73.3) | 7/13 (53.8) | 0.249 |  |
| Effectiveness of hormone therapy, n (%) | 36/46 (78.3) | 17/20 (85.0) | 19/26 (73.1) | 0.273 |  |
| History of cesarean section, n (%) | 31 (39.2) | 14 (41.2) | 17 (37.8) | 0.759 | 0.867(0.349-2.157) |
| History of diagnostic curettage, n (%) | 28 (35.4) | 11 (32.4) | 17 (37.8) | 0.618 | 1.269 (0.497-3.243) |
| Concurrent leiomyoma (Pathology), n (%) | 46 (58.2) | 21 (61.8 | 25 (55.6) | 0.580 | 0.774(0.312-1.918) |
| Endometrium status (Pathology), n (%) |  |  |  |  |  |
| Proliferative phase | 54 (68.4) | 24 (70.6) | 30 (66.7) | 0.711 |  |
| Secretory phase | 7(8.9) | 5 (14.7) | 2 (4.4) | 0.118 |  |
| Atrophic/Quiescence | 9 (11.4) | 3 (8.8) | 6 (13.3) | 0.400 |  |
| Endometriosis (Pathology), n (%) | 34 (43.0) | 16 (47.1) | 18 (40.0) | 0.530 | 0.750(0.305-1.844) |
| Ovarian type | 18 (22.8) | 6 (17.6) | 12 (26.7) | 0.344 | 1.697 (0.564-5.107) |
| DIE | 16 (20.3) | 8 (23.5) | 8 (17.8) | 0.529 | 0.703 (0.234-2.113) |
| Peritoneal type | 19 (24.1) | 9 (26.5) | 10 (22.2) | 0.662 | 0.794 (0.281-2.238) |

Abbreviation: BMI, Body mass index; NA, not available; GnRHa, gonadotrophin releasing hormone agonist; DFI, disease free interval; DIE, deep invasive endometriosis; WGD, whole genome doubling; SD, standard deviation.

# Supplementary Table 6. Multivariate analysis of the early onset of adenomyosis.

| Parameter | β | S.E. | *P* value | OR (95% CI) |
| --- | --- | --- | --- | --- |
| WGD existing | 3.839 | 1.007 | **<0.001** | 46.485(6.454-334.811) |
| Height (cm), median (range) | 0.120 | 0.075 | 0.108 | 1.128 (0.974-1.306) |
| BMI (kg/m^2^), median (range) | 0.011 | 0.090 | 0.899 | 1.011 (0.849-1.205) |
| Parity (times), median (range) |  |  |  |  |
| 0 |  |  | 0.260 |  |
| 1 | -2.107 | 1.051 | **0.045** | 0.122 (0.015-0.955) |
| 2 | -1.772 | 1.355 | 0.191 | 0.170 (0.012-2.419) |
| 3 | 19.258 | 40192.969 | 1.000 | 2.3*10^8^ (0-∞) |
| Dysmenorrhea | 0.649 | 0.891 | 0.467 | 1.913 (0.334-10.964) |
| Vaginal bleeding, n (%) | -0.471 | 0.759 | 0.534 | 0.624 (0.141-2.761) |
| History of cesarean section, n (%) | 1.717 | 0.827 | **0.038** | 5.565(1.100-28.157) |
| History of diagnostic curettage, n (%) | 0.384 | 0.648 | 0.553 | 1.468 (0.413-5.223) |
| Endometriosis (Pathology), n (%) |  |  |  |  |
| Ovarian type | -2.337 | 0.978 | **0.017** | 0.097 (0.014-0.658) |
| DIE | 0.084 | 0.840 | 0.920 | 1.088 (0.210-5.641) |
| Peritoneal type | 1.283 | 0.779 | 0.100 | 3.607 (0.783-16.618) |
| Intercept | -19.856 | 11.963 | 0.097 |  |

Abbreviations: NA, not available; GnRHa, gonadotrophin releasing hormone agonist; DFI, disease free interval; DIE, deep invasive endometriosis; WGD, whole genome doubling; SD, standard deviation.

# Supplementary Table 7. The sample distribution of SNV and driver genes.

| Gene mutation | WGD samples | non-WGD samples | *P* value |
| --- | --- | --- | --- |
| Driver genes_yes | 7 | 75 | 0.07926 |
| Driver genes_no | 26 | 126 |  |
| SNV_yes | 30 | 171 | 1 |
| SNV_no | 3 | 20 |  |

# Supplementary Table 8. The distribution of patients with *KRAS* mutation.

| Patients | *KRAS* mutation | *KRAS* wild type | *P* value |
| --- | --- | --- | --- |
| AM (n=79) | 19 (24.1%) | 60 (75.9%) | 0.09687 |
| EM (n=22) | 1 (4.5%) | 21 (95.5%) |  |
| Controls (n=20) | 3 (15.0%) | 17 (85.0%) |  |

# Supplementary Table 9. The distribution of patients with *PIK3CA* mutation.

| Patients | *KRAS* mutation | *KRAS* wild type | *P* value |
| --- | --- | --- | --- |
| AM (n=79) | 15 (19.0%) | 64 (81.0%) | 0.2494 |
| EM (n=22) | 2 (9.1%) | 20 (90.9%) |  |
| Controls (n=20) | 1 (5.0%) | 19 (95.0%) |  |

# Supplementary Table 10. The relationship between *KRAS* and *PIK3CA* mutations and effectiveness of endocrine therapy.

| Gene mutations | Effectiveness | ineffectiveness | *P* value |
| --- | --- | --- | --- |
| *KRAS* mutation | 4 | 3 | 0.686 |
| *KRAS* wild type | 25 | 13 |  |
| *PIK3CA* mutation | 3 | 5 | 0.111 |
| *PIK3CA* wild type | 26 | 11 |  |

Note: The effectiveness of hormone therapy for AM before surgery was determined by the improvement of uterine bleeding or painess or uterine volume decreasing.

# Supplementary Figure 1. Proportions of the genome subject to loss of heterozygosity (LOH) and haploid LOH in WGD and nWGD samples.

# Supplementary Figure 2. The mutual exclusion and co-occurrence relationship between driver genes (A), and GO and KEGG pathway enrichment analysis of 33 differential CNV loss genes performed by Metascape (B).

# Supplementary Figure 3. Comparison of variant allele frequency (VAF) of *KRAS* and *PIK3CA* genes among AM patients, EM patients and controls groups. For multiple-lesion patients, variants detected in more than one sample, VAF take the maximum as the measured value based on WES.
